# Acute Intussusception. II

**Published:** 1894-06-16

**Authors:** W. McAdam Eccles

**Affiliations:** Assistant Surgeon to the West London Hospital, Assistant Demonstrator of Anatomy at St. Bartholomew's Hospital, Assistant Surgeon to the City of London Truss Society, and Surgeon to the St. Marylebone General Dispensary


### ACUTE INTUSSUSCEPTION.—II.

By W. McADAM ECCLES, M.B., B.S.(Lond.), F.R.C.S. Eng., Assistant Surgeon to the West London Hospital, Assistant Demonstrator of Anatomy at St. Bartholomew's Hospital, Assistant Surgeon to the City of London Truss Society, and Surgeon to the St. Marylebone General Dispensary.

**The diagnosis** of cases of acute intussusception of the intestine is generally clear, and early recognition of this disease is most desirable. As has already been noted, if a child without any strangulated hernia be suddenly seized with signs of acute intestinal obstruction, the condition in a very large number of cases is due to the presence of an acutely invaginated bowel, and if one or more of the cardinal symptoms—viz., abdominal pain, vomiting, the passage of blood-stained mucus, and the feeling of a tumour—be in evidence the diagnosis is practically certain.

Acute gastro-enteritis may sometimes rather closely simulate intussusception, but the sudden onset, the patient being often apparently quite well till the beginning of the symptoms, a tumour to be made out, and the rapid exhaustion, all point to invagination rather than inflammation. Prolapse of the rectum might, without careful examination, be mistaken for the projection of an intussusception, or *vice versa.*

The presence of a polypus in the rectum I have known lead to an erroneous diagnosis of intussusception. A digital examination of the rectum would probably have cleared up the condition.

The **prognosis** of acute invagination is highly unfavourable. The mortality very markedly increases with the length of time from the commencement of the symptoms.

In a recent analysis of twenty-eight cases which I made (see St. Bartholomew's Hospital Reports, vol. xxviii., p. 97), one case which was treated on the first day of the disease recovered; of six seen on the second day four lived and two died; of eight admitted on the third day no less than six terminated fatally, only two recovering, and of those admitted later all but two perished.

Thus it would appear that the prognosis may with fairness be judged by the duration of the disease.

Again, the age of the patient has an important bearing upon the result, it almost being needless to remark that the younger the child the graver the prognosis.

The acuteness of the attack will also tend to a rapidly fatal result; in fact, some cases terminate within a few hours. And, lastly, the method of treatment will, without doubt, modify very considerably the question of prognosis. This all-important portion of the subject will now be dealt with.

**Treatment of Cases of Acute Intussusception.**—In the paper quoted above, I concluded by saying that for the successful treatment of cases of intussusception, early diagnosis of the disease is of the utmost importance, and that any child with one or both of the important signs or symptoms, namely, the passage of blood and mucus per rectum, and the presence of a tumour felt per rectum or per abdomen, should at once be treated as if it were a case of invaginated gut, and I think this statement is justifiable in view of the results of such a line of conduct. The treatment of intussusception is the subject of a very large amount of literature, and after reading the bulk of this, and having observed a considerable number of cases, I have come to the conclusion that the practical treatment of this disease resolves itself into three methods:—

1. That of leaving the patient alone, beyond cautiously giving opium, or opium and belladonna, by the mouth or hypodermically, and hoping for the very slender chance of a spontaneous cure by the sloughing off and evacuation of the intussusception. This line of action I would most strongly condemn, except of course in cases which when seen are practically hopeless, or at any rate too advanced apparently to do any good to by attempting an operation.

2. That of trying to reduce the gut by means of the distension of the bowel below the invagination, either with liquid or gas, and helped in some cases by manipulation through the abdominal walls. I shall discuss this under three practical headings:—(*a*) Cases suitable for this line of treatment. (i.) They must be recent. It will be but rarely successful, I am disposed to believe, after forty-eight, or at the most fifty-four hours from the commencement of the acute symptoms. (ii.) It is not satisfactory to employ it unless a definite tumour has been observed, otherwise it would be extremely difficult to be sure of the effect of the injection, at any rate immediately. (iii.) It would hardly be likely to be of any benefit if the invaginated bowel were already in the rectum. (*b*) The choice between gas and liquid. I am strongly in favour of the latter, for the following reasons: (i.) Liquid is much more easily introduced into, and retained within the bowel. (ii.) It can be injected with great exactness as regards force and quantity. (iii.) Its greater density will probably have some action in more effectively reducing the gut. (iv.) That the statistics show a decidedly better result with liquid than with gas. If gas be chosen, what gas is used is quite a minor detail. Lately a successful case of reduction has been recorded, in which carbonic acid gas was generated within the bowel by the action of acid upon a carbonate. If liquid be decided upon it may be water, oil, milk, or gruel, of which I think milk is as good as any, if it be at hand. (*c*) The method of injection: (i.) The liquid should be of the temperature of 99 deg. F. while being injected. (ii.) The child should always, if possible, be anæsthetised with chloroform. This, I believe, is a very important detail, for not only does it efface pain, but allows a much greater quantity of fluid to be injected, and what is of prime importance, allows the surgeon to easily notice and feel the effect of the injection upon the tumour. (iii.) The liquid may either be introduced with a Higginson's syringe, or by hydrostatic pressure. The latter is the better way, the reservoir not being raised higher than three feet. (iv.) The child may be partially or wholly inverted during the process, and manipulation may be applied through the abdominal walls. (v.) The patient must be protected against exposure. (vi.) Small doses of opium should be administered subsequently to reduction.

A word of warning must here be given. In many cases the tumour is apt to become impalpable, owing to its having disappeared up under the liver or elsewhere, and this must not be mistaken for its complete reduction. Often the condition of invagination is said to recur after reduction by fluid. I believe most of such cases are really instances of incomplete restoration of the bowel to its normal condition, for the last portion of the intussusceptum is often very difficult to reduce, and may be the starting point for a relapse into a state quite as bad as before.

(3). Lastly, we have the treatment by laparotomy and so reducing the invagination by direct manipulation; or, if this be impossible, of otherwise dealing with the gut. If one careful and thorough attempt at reduction by injection fail to completely push back the gut, a fact which is often uncertain but evidenced either by the continuation of the symptoms or better the presence of the tumour, I feel sure the best plan is, without further delay, to open the abdomen as a rule. In performing laparotomy under these circumstances the following points should be borne in mind: (i.) To operate promptly, but not hurriedly. (ii.) To very carefully protect the young patient from all unnecessary exposure during the operation by keeping the limbs wrapped in cotton wool and having the infant laid upon a hot-water cushion. (iii.) The incision is probably best commenced in the middle line above the umbilicus, but may have to be enlarged downwards. (iv.) As soon as the abdomen has been opened the fingers of the operator should be inserted and the tumour felt for, and brought to the surface if possible. (v.) Reduction should then be effected by squeezing the gut out rather than by dragging, though the latter may be gently employed. In a large number of cases, especially if early, the gut is easily reduced, except perhaps the very last portion, chiefly in the ileo-cæcal variety. This is due to the highly œdematous condition of the part. Firm pressure around the circumference of the gut with the hand might aid in freeing this portion. (vi.) When reduction has been brought about, any escaped small intestine will have to be returned, and this is most easily accomplished by the edges of the abdominal wound being held up, and so to speak being brought over the gut, rather than the bowel being forced back into the abdomen. Great care must be taken that any exposed intestine is not chilled. (vii.) The wound is now rapidly closed, and the child returned to a thoroughly warmed bed in front of a fire, and minute doses of opium given every few hours. (viii.) If reduction cannot be effected, probably the best line of treatment is to establsh an artificial anus by stitching and opening the bowel above the invagination. Such cases, however, usually end fatally.

The results of laparotomy even in very young subjects have of late years been highly encouraging, but it still remains for every practitioner to diagnose his cases early, and then to treat them as above indicated promptly if he would have good success.

I am sure the day is not far distant when the mortality of over 70 per cent. of acute cases will be reversed, with a percentage of recoveries exceeding our most sanguine anticipations.

